# Frequency of the Apolipoprotein E ε4 Allele in a Memory Clinic Cohort in Beijing: A Naturalistic Descriptive Study

**DOI:** 10.1371/journal.pone.0099130

**Published:** 2014-06-10

**Authors:** Xiao Wang, Huali Wang, Huiying Li, Tao Li, Xin Yu

**Affiliations:** 1 Dementia Care and Research Center, Peking University Institute of Mental Health (Sixth Hospital), Beijing, China; 2 Key Laboratory for Mental Health (Peking University), Ministry of Health, Beijing, China; 3 Chaoyang District Third Hospital, Beijing, China; Beijing Normal University, Beijing, China

## Abstract

**Objectives:**

To describe the distribution of apolipoprotein E (APOE) genotypes among an elderly Chinese patient population with memory complaints treated in a memory clinic in Beijing and to compare the ε4 allele frequency among individuals with subjective cognitive impairment (SCI), mild cognitive impairment (MCI) and Alzheimer's disease (AD).

**Methods:**

A total of 385 subjects with memory complaints participated in the study, including 216 patients with AD, 56 with MCI, 17 with SCI, and 96 with other types of cognitive impairment. A total of 75 healthy elderly control subjects were also recruited. The polymerase chain reaction-restriction fragment length polymorphism (PCR-RFLP) technique was used to investigate the APOE genotypes.

**Results:**

The frequency of the ε4 allele was 19.6 percent for the entire sample of patients who had memory complaints. The APOE allele distribution differed between women and men (22.6% and 14.9%, respectively; *p*<0.05) in the individuals with memory complaints. Compared with the control group (7.3%), the prevalence of the APOE ε4 allele was significantly higher in the AD (23.6%) and MCI (21.4%) groups and was slightly increased in the SCI (14.7%) group.

**Conclusions:**

In the memory clinic, we observed a higher prevalence of the APOE ε4 allele among Chinese AD and MCI patients. A similar trend was observed in patients with SCI. These findings suggest that nondemented APOE ε4 allele carriers with memory complaints may have a greater genetic risk for AD and should be monitored more closely.

## Introduction

The apolipoprotein E (APOE) ε4 allele is the most established genetic risk factor for sporadic Alzheimer's disease (AD) [1], [2]; its presence increases the risk for developing AD at a younger age in a gene-dose dependent manner [3]. Previous neuropsychological studies have shown that patients with AD [4]–[10] and mild cognitive impairment (MCI) [11] who are APOE ε4 carriers perform worse on memory tasks. Several previous studies have also reported that the prevalence of the APOE ε4 allele was higher among patients with MCI, AD and other types of dementia compared with the general population [12], [13]. In addition, the scientific literature has demonstrated that the APOE ε4 genotype is associated with a more rapid progression from MCI to AD [14]–[16]. Healthy elderly patients with subjective cognitive impairment (SCI) who do not show any evidence of cognitive impairment on formal testing [17] are 4.5 times more likely to progress to the more advanced memory loss stages of MCI or dementia than patients without SCI [18], [19]. It is estimated that approximately one third of patients visiting memory clinics have SCI [20]; a higher proportion of these patients carry the APOE ε4 allele versus other forms of the APOE allele [21], [22]. Therefore, it is extremely important to identify potential risk factors for the future development of AD so that these patients can receive closer monitoring in memory clinics.

Previous community-based studies have found that Chinese patient populations possess a relatively lower frequency of the APOE ε4 allele [23], [24]; hospital-based case studies have also observed a similar distribution of APOE genotypes in AD patients [25]. A recent pilot study in Shanghai showed associations between MCI and AD and the APOE ε4 and ε2 alleles in a small sample [13]. Nevertheless, these previous studies did not include patients with SCI; memory clinic-based studies may be more representative and have more important clinical implications.

Memory clinics offer specialized services for people with memory loss and dementia. In these clinics, diagnostic and interventional services are provided to people who are concerned about changes in their memory or the memory of someone they care for. During the past two decades, memory clinics have been developed in several cities across China and have become the primary setting in which people with memory complaints seek care. Most commonly, highly trained neurologists and geriatric psychiatrists are the primary personnel who direct the memory clinics in China. In particular, these specialists provide an early and accurate diagnosis of dementia and identify individuals at risk for the future development of dementia. However, little is known about the distribution of APOE genotypes among the elderly Chinese patient population who seek help at these memory clinics.

Therefore, we designed this prospective study primarily to describe the distribution of APOE genotypes among patients with memory complaints in our memory clinic. Second, we compared the frequency of APOE alleles among patients with AD, MCI, and SCI as well as a healthy control group. Based on previous reports [12], we hypothesized that the frequency of the APOE ε4 allele will be increased in the MCI and AD groups and that the ε4 allele in the SCI group will show an intermediate frequency somewhere between the levels observed in the MCI and control groups, indicating that elderly patients with SCI may be predisposed to develop AD in the future.

## Materials and Methods

This study protocol was approved by the institutional review board of the Peking University Institute of Mental Health (Sixth Hospital). Written informed consent for participation in the case registry was obtained from each subject.

### Subjects

All individuals who participated in the case registry were diagnosed with thorough clinical work-ups that included a documentation of their medical and family history, physical and neurological examinations, neuropsychological assessment, multi-modal neuroimaging check-ups (details as previously described [26]), laboratory investigations (including complete blood count, electrolytes, blood chemistry, thyroid function testing, vitamin B_12_ and folic acid levels, syphilis detection values), electrocardiography (ECG), and an optional blood donation for genetic sub-studies. Each subject may have also opted to consent to participate in the longitudinal observational sub-study. Specifically, all of the patients with MCI and AD received a clinical examination, battery of neuropsychological tests (including a mini-mental status examination (MMSE) [27], [28] and a neuropsychiatric evaluation), and routine laboratory tests approximately every 6 months. The cognitively normal elderly participants, including normal controls and individuals with SCI, underwent the same examinations and tests on an annual basis. All subjects received an annual multi-modal brain MRI scan, including 3D volumetric MRI, diffusion tensor imaging and resting-state functional MRI.

**Figure 1:**
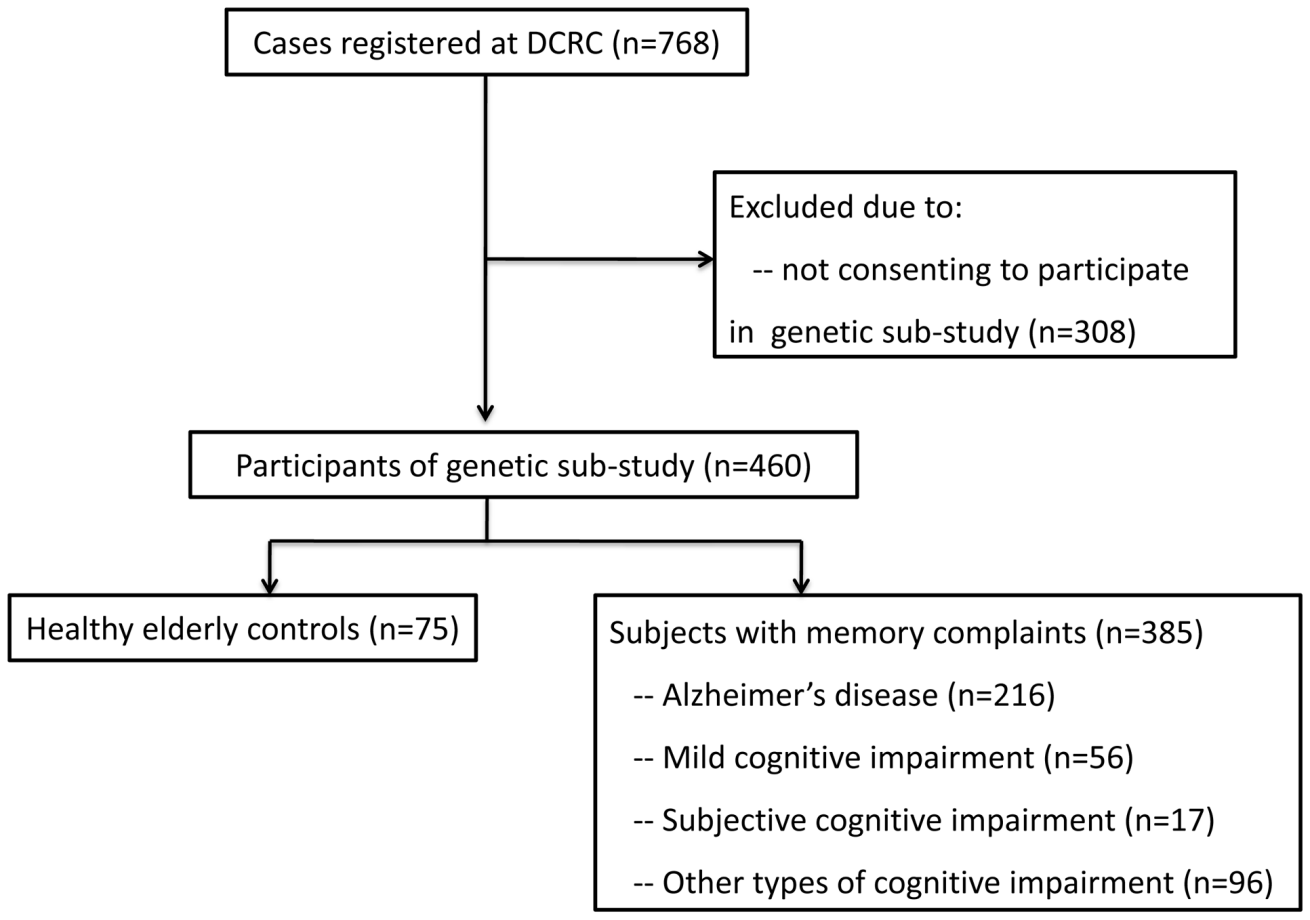
Flow chart of subject recruitment and selection for the genetic sub-study.

As summarized in the subject recruitment flow chart (Figure 1), 768 participants of unrelated Han Chinese descent were prospectively registered at the Dementia Care & Research Center of the Peking University Institute of Mental Health from February 2006 to January 2010. Among these participants, 385 with memory complaints (151 men and 234 women; mean age 73.2±8.6 years; ranging from 42 to 98 years) consented to participate in the optional genetic sub-study, donated their whole-blood sample and were included in this study. Among these participants, 216 patients had AD, 56 had MCI, 17 had SCI, and 96 had other types of cognitive impairment; 75 healthy elderly controls were also recruited. One 42-year-old patient with memory complaints was recruited and diagnosed with neurosyphilis upon the first visit to our memory clinic. The clinical diagnosis of AD was made according to the criteria for dementia in the International Classification of Diseases, 10^th^ Revision (ICD-10) [29] and the criteria for probable AD of the National Institute of Neurological and Communicative Disorders and the Stroke/Alzheimer Disease and Related Disorders Association (NINCDS-ADRDA) [30]. The clinical diagnosis of MCI was made according to the criteria established by Petersen [31]. Other types of cognitive impairment included in this study were all other types of dementia, such as vascular dementia [32], Lewy body dementia [33] and frontotemporal dementia [34]. The individuals who did not fulfill these criteria for dementia or any of the other diagnostic categories but did complain of memory decline were considered to have SCI. The scores on the MMSE and the clinical memory scale (CMS) [35] in participants with SCI were within the age-, gender- and education-adjusted norms and their social and daily functioning was intact. The healthy control subjects had no complaints of cognitive problems and showed no evidence of cognitive deficits on neuropsychological testing.

### APOE genotyping

Blood samples used for genotyping were collected in ethylenediaminetetraacetic acid (EDTA)-containing receptacles. DNA was extracted from peripheral blood using the QIAamp DNA Blood Mini Kit (Qiagen Inc., Hilden, Germany). To determine the APOE genotype of the participant (APOE ε2, APOE ε3, APOE ε4 allele), the polymerase chain reaction-restriction fragment length polymorphism (PCR-RFLP) technique, as originally developed by Emi [36] and Hixson [37], was used. The primers used were 5′-ACAGAATTCGCCCCGGCCTGGTACAC-3' (sense) and 5′-TAAGCTTGGCACGGCTGTCCAAGGA-3′ (antisense) [1]. The reaction mixture (total volume of 15 µl) was prepared with 0.5 µl of each primer, 0.5 µl of dNTPs, 1 U of Taq polymerase (Takara Bio Inc., Otsu, Japan), 7.5 µl of 2×GC buffer, and 100 ng of DNA from the sample. The amplification process (Applied Biosystems 2720 Thermal Cycler, Applied Biosystems Inc., Carlsbad, USA) consisted of an initial denaturation at 94°C for 5 min, followed by 40 cycles of denaturation at 94°C for 1 min, coiling at 63.8°C for 1 min, and extension at 72°C for 30 sec, and a final extension at 72°C for 7 min. The efficacy of the PCR assay was visualized through electrophoresis on a 1.5% agarose gel with ethidium bromide; the procedure was effective when a 244-bp band was observed under ultraviolet light. Then, the PCR amplification products were digested with 10 U of H*haI* endonuclease (Fermentas International Inc., Ontario, Canada) at 37°C overnight. Electrophoresis was performed in a TBE buffer. The gel concentration and running conditions were as follows: 12% polyacrylamide, 3 h, room temperature, 200 V. After electrophoresis, the gels were stained with ethidium bromide. The APOE genotypes of the samples were deduced from the observed combinations of different-sized fragments. Genotype scorers (authors Wang X and Li H) were blinded to the identity of the samples. Eighteen samples were selected to be sequenced using a gene sequencing technique. The sequencing results further verified the APOE genotyping results based on the PCR-RFLP approach.

### Statistical analysis

Statistical analyses were performed using the Statistical Package for Social Studies (SPSS) version 13.0. The frequencies of the APOE alleles were obtained using SHEsis Online Version (available at http://analysis.bio-x.cn/SHEsisMain.htm). All continuous variables are expressed as the mean ± standard deviation (SD). Differences in the APOE allele carriage and sex were evaluated with the chi-squared test. Within the diagnostic categories, group comparisons of continuous variables according to their APOE ε4 status were performed using one-way ANOVA. We computed Pearson's correlation coefficient between the APOE ε4 status and MMSE score in each of the diagnostic categories. The significance level was defined as α = 0.05.

## Results

Among the entire sample of patients with memory complaints in this study, 234 (60.8%) were female, and the mean age was 73.2±8.6 years (ranging from 42 to 98 years). There were 216 (56.1%) patients with AD, 56 (14.5%) with MCI, 17 (4.4%) with SCI, and 96 (24.9%) with other types of cognitive impairment, including mixed dementia (n = 6), Binswanger's disease (n = 1), cardiovascular disease (n = 1), vascular cognitive impairment (n = 4), vascular dementia (n = 1), Lewy body dementia (n = 8), frontotemporal dementia (n = 14), Parkinson's disease with dementia (n = 2), neurosyphilis (n = 1), mood disorder with or without cognitive impairment (n = 55), and others (n = 3).

Table 1 shows the demographic characteristics of the participants according to diagnostic group (i.e., AD, MCI, SCI and control groups). The age at first visit (in years) to the memory clinic was higher in the AD group compared with the control group (*p*<0.01). The gender distribution differed between the control group and the AD and SCI groups (AD vs. control, χ^2^ = 13.76, *p*<0.001; SCI vs. control, χ^2^ = 4.8, *p* = 0.029). The patients in the AD group received fewer years of education compared with all other diagnostic groups (all *p*<0.05). The mean MMSE score was lower in the AD group than in all other groups (all *p*<0.05) as well as in the MCI group compared with the control group (*p* = 0.033, see Table 1).

**Table 1:** Demographic characteristics of the AD, MCI, SCI and control groups.

	AD (n = 216)	MCI (n = 56)	SCI (n = 17)	Controls (n = 75)
Age at first visit (years)	75.6±7.9*	73.3±7.5	72.2±6.5	71.5±8.8
Gender (men/women)	74/142*	24/32	5/12*	44/31
Education (years)	11.2±5.4* ^,#,^§	14.0±3.3	14.6±3.5	13.8±3.5
MMSE score	16.9±6.4* ^,#,^§	26.5±3.1*	28.9±1.2	28.5±1.5

*p<0.05, vs. control group; #*p*<0.05, vs. MCI group; ^§^ *p*<0.05 vs. SCI group.

Among the 385 subjects with memory complaints, the frequency of the APOE ε4 allele was 19.6%, and the APOE ε4/ε4 genotype was observed in 18 subjects (4.7%). Compared with the control group (7.3%), the prevalence of the APOE ε4 allele was higher among the individuals with memory complaints (χ^2^ = 13.04, *p*<0.01). As shown in Table 2, the distribution of APOE genotypes and alleles was significantly different between men and women (*p* = 0.027). The ε4 allele frequency was remarkably greater in women compared with men (22.6% in women vs. 14.9% in men, χ^2^ = 6.99, *p* = 0.008). More APOE ε4 homozygous carriers were observed in women compared with men (6.4% in women vs. 2.0% in men, χ^2^ = 4.03, *p*<0.05). However, the differences in the frequency of the APOE ε4 allele and the percentage of APOE ε4 homozygous carriers were not different across age groups (*p*>0.05).

**Table 2:** Frequency of APOE genotypes and alleles among participants with memory complaints stratified by gender and age.

		Frequency of APOE alleles (%)			Frequency of APOE genotypes (N, %)					
	N	ε2	ε3	ε4	ε2/ε2	ε2/ε3	ε2/ε4	ε3/ε4	ε3/ε3	ε4/ε4
Gender										
Men	151	6.6	78.5	14.9	5(3.3)	7(4.6)	3(2.0)	36(23.8)	97(64.2)	3(2.0)
Women	234	6.8	70.5	22.6*	7(3.0)	11(4.7)	7(3.0)	69(29.5)	125(53.4)	15(6.4)*
Age at first visit (years)										
<60	35	2.9	74.3	22.9	0(0.0)	2(5.7)	0(0.0)	8(22.9)	21(60.0)	4(11.4)
60–69	71	9.2	75.4	15.5	3(4.2)	2(2.8)	5(7.0)	17(23.9)	44(62.0)	0(0.0)
70–79	187	5.6	73.5	20.9	4(2.1)	10(5.3)	3(1.6)	61(32.6)	102(54.5)	7(3.7)
≥80	92	8.7	75.0	16.3	5(5.4)	4(4.3)	2(2.2)	24(26.1)	55(59.8)	2(2.2)
Total	385	6.8	73.6	19.6	12(3.1)	18(4.7)	10(2.6)	105(27.3)	222(57.7)	18(4.7)

*p<0.05, women vs. men.

Table 3 shows the APOE allele and genotype frequencies for the 4 diagnostic groups. The AD and MCI groups showed a greater frequency of the APOE ε4 allele compared with healthy controls (23.6% for AD and 21.4% for MCI vs. 7.3% for control; AD vs. control, χ^2^ = 18.86, *p*<0.01; MCI vs. control, χ^2^ = 11.01, *p* = 0.001). There was no significant difference in the frequency of the APOE ε4 allele between the AD and MCI groups (23.6% for AD vs. 21.4% for MCI, χ^2^ = 0.24, *p*>0.05). The frequency of the APOE ε4 allele in the SCI group showed a trend toward being an intermediate level between the AD/MCI and healthy control groups (SCI: 14.7%, AD: 23.6%, MCI: 21.4%, and controls: 7.3%). However, this slight difference did not reach the level of significance (see Table 3).

**Table 3:** Comparison of the frequencies of APOE genotypes and alleles among the AD, MCI, SCI and control groups.

		Frequency of APOE alleles (%)			Frequency of APOE genotypes (N, %)					
	N	ε2	ε3	ε4	ε2/ε2	ε2/ε3	ε2/ε4	ε3/ε4	ε3/ε3	ε4/ε4
AD group	216	6	70.4	23.6*	4(1.9)	12(5.6)	6(2.8)	66(30.6)	113(52.3)	15(6.9)*
MCI group	56	8.9	69.6	21.4*	3(5.4)	3(5.4)	1(1.8)	23(41.1)	26(46.4)	0(0.0)
SCI group	17	2.9	82.4	14.7	0(0.0)	0(0.0)	1(5.9)	4(23.5)	12(70.6)	0(0.0)
Control group	75	11.3	81.3	7.3	1(1.3)	12(16)	3(4.0)	8(10.7)	51(68.0)	0(0.0)

*p<0.01, vs. control group.

Table 4 shows the mean MMSE score according to the APOE ε4 status for each diagnostic group. No significant correlations were found between the MMSE score and the APOEε4 status in any of the diagnostic groups (all *p*>0.05).

**Table 4:** MMSE score by number of APOE ε4 alleles in the four diagnostic groups.

	No ε4 allele	1 ε4 allele	2 ε4 allele
AD group (N, %)	129(60)	72(33)	15(7)
MMSE	17.4±6.4	15.7±6.3	17.8±5.8
MCI group (N, %)	32(57)	24(43)	0
MMSE	26.3±3.6	26.7±2.5	-
SCI group (N, %)	12(71)	5(29)	0
MMSE	29.3±0.6	28.2±2.0	
Control group (N, %)	64(85)	11(15)	0
MMSE	28.5±1.5	28.7±1.2	-

## Discussion

In our memory clinic cohort, the frequency of the APOE ε4 allele among individuals with memory complaints was 19.6%, significantly higher than in normal controls (7.3%), indicating that people with memory complaints may be at an increased risk for AD; however, some of these individuals may maintain cognitive performance that is comparable to healthy controls. The ε4 allele frequency among the normal controls in our sample was lower than the frequency previously reported in Caucasian individuals [12] but was similar to a recent report from a Shanghai memory disorder clinic, where the frequency detected was 5% [13]. The higher percentage of the ε4 allele in our sample with memory complaints may be because a majority of visitors to our memory clinic are AD and MCI patients.

We observed that among individuals with memory complaints, the frequency of the ε4 allele differed between women and men. This result may partially contribute to the difference in the prevalence of AD observed between men and women [38]. Previous studies reported that women are more likely than men to develop AD across most age groups [39], [40]; this finding may be related to the decline of estrogenic mediators in older women [40]. The studies on the APOE gene in AD have also shown that the ε4 allele is more pronounced in women [39], [41], [42], which might explain the higher amount of amyloid plaques and neurofibrillary tangles observed in female ε4 carriers [43].

As observed in earlier studies on memory clinic cohorts [12], we did not observe an association between the APOE ε4 allele frequency and the age at first visit within our sample of patients with memory complaints. Although it may be meaningful to explore the effect of the ε4 allele on the age at onset, precisely defining the age at onset is impractical because AD and MCI usually have an insidious onset with a gradual progression, and symptoms may often be ignored for several years.

This study, which included a relatively large cohort of individuals being observed at a memory clinic, confirmed that the prevalence of the APOE ε4 allele is higher among AD patients relative to healthy controls [12], [44], [45]. This study also confirmed that a high ε4 allele frequency is observed in AD within non-Caucasian populations in a similar proportion as that observed in Caucasian populations [12]. The frequencies of the ε2, ε3, and ε4 alleles in our AD sample were 6%, 70.4%, and 23.6%, respectively; these measurements are very similar to the frequencies reported in previous studies conducted in Chinese patient populations [25]. The APOE ε4 allele is considered to be one of the major genetic risk factors for AD [1] and has been associated with cognitive decline in neuropsychological studies [46], [47]. Additionally, a higher ε4 allele frequency was observed among individuals with MCI. The ε4 allele frequency was comparable between the AD and MCI groups in our study. The role of the ε4 allele on MCI among Chinese elderly patients is inconsistent in earlier studies conducted in Shanghai (30 aMCI vs. 32 controls) [13], Hefei (28 MCI vs. 30 controls) [48] and Taiwan (58 MCI vs. 20 controls) [49]. Our results are in agreement with those studies that reported higher a prevalence of the ε4 allele in MCI, implying that MCI may have a greater genetic risk for the future development of AD [13], [48], [49]. However, different diagnostic criteria for MCI may have been applied in each of these studies, which may explain the differences observed in the estimates for the APOE genotypes between studies [50].

No significant correlations were found between the MMSE scores and the APOE ε4 status in any of the diagnostic groups, which is consistent with a previous study [12]. Several previous studies have suggested that the APOE ε4 allele is associated with a faster rate of cognitive decline, rather than cognitive impairment, at the first visit [22], [51]. However, other studies did not detect an effect of the APOE ε4 allele on the rate of cognitive decline [52], [53].

Interestingly, our study demonstrated that the frequency of the ε4 allele among individuals with SCI was slightly greater than in normal controls but lower than in patients with AD and MCI. Although the trend did not reach statistical significance, the result implies that subjective complaints, even in the absence of objective cognitive impairment, may be a harbinger to further cognitive decline [18]. The marginal increase in the ε4 allele frequency may result in a higher genetic risk for the future development of AD. However, only 17 cases of SCI were included in our study; therefore, such a conclusion would be premature. Clinical follow-up of the present cohort is necessary to provide actual risk estimates for dementia associated with the APOE ε4 allele in the SCI and MCI groups.

The findings of our memory clinic cohort study may have significant clinical practice implications. By raising public awareness of dementia, memory clinics have become widespread and provide specialized services for people with memory problems. Patients with memory complaints come to clinicians with the belief that they, or a family member they care about, may have a diagnosable problem. Clinicians in these memory clinics are usually asked the following questions: “Is this a problem?” or “Will I (or he/she) develop dementia?” The findings of this study indicate that compared with the general population, APOE ε4 carriers may be predisposed to attend memory clinics when they are suffering from memory complaints. These findings also remind clinicians to be attentive to memory clinic visitors and to keep APOE ε4 carriers in an intensive monitoring program. This type of monitoring is particularly important in China, where the medical referral system has not been fully established and individuals living in the community usually seek medical care according their personal preferences.

The relatively limited number of individuals with SCI may be one of the major disadvantages of our study; however, this small proportion likely illustrates the low level of public awareness regarding pre-dementia states. Additionally, the cross-sectional nature of our study limits the conclusion concerning the association between the APOE ε4 allele and both MCI and SCI as well as the risk for developing AD. Our study was conducted at a well-established memory clinic in Beijing; the comprehensive clinical work-ups implemented among memory clinic attendees in this study may be one of the strengths of this study. However, due to inequalities in the service provision of and access to dementia care across the nation, our findings may not reflect the full pattern of distribution of diagnostic groups in memory clinics nationwide. Therefore, further longitudinal and multi-center prospective case registry studies are warranted.

In conclusion, the frequency of the APOE ε4 allele among AD and MCI patients was higher than among healthy controls in our memory clinic cohort. This finding implies that APOE ε4 carriers with memory complaints may be more likely to visit memory clinics. Clinically, follow-up observations should be implemented for the timely detection of further progressive cognitive deterioration.
